# Data on Spectroscopic, Rheological characterization of neem oil and its isolated fractions

**DOI:** 10.1016/j.dib.2018.10.049

**Published:** 2018-10-25

**Authors:** Samir Bhargava, N.V. Satheesh Madhav

**Affiliations:** a DIT Faculty of Pharmacy, DIT University, Dehradun, Uttarakhand 248001, India; b Faculty of Pharmacy, Uttarakhand Technical University, Dehradun, Uttarakhand 248001, India

**Keywords:** Neem oil, Neem oil fractions, Gas chromatography, Rheology

## Abstract

In this article a medicinal oil (neem oil) is fractionated and compared with original oil. The fractions were separated at low temperature using chloroform and methanol. The uniphase mixture of solvents and neem oil at room temperature was transformed to a bi-phasic system at low temperature. The isolated fractions (NOC – isolated using chloroform; NOM – isolated using methanol) were characterized and differentiated by GC, FT-IR and Rheometer. GC and FTIR have well revealed the difference in composition of fatty acids in fractions – NOC; NOM and neem oil (NO). Rheologically all the oils are different in viscosity from parent oil. The NOM fraction of neem oil showed newtonian behavior while NOC shows a non-newtonian behavior. It can be concluded from data that fractions NOC, NOM can be used for targeting drugs using various formulation approaches.

## Specifications table

**Table:** 

Subject area	Chemistry
More specific subject area	Lipids and Biology.
Type of data	Table, Figure and Graph
How data was acquired	GC (Thermo Trace 1300GC), IR (Shimadzu FT IR Infinity-1), Rheometer (Anton Par MCR 72)
Data format	Raw and analyzed
Experimental factors	Fractions of oil have been isolated using different solvents
Experimental features	Solubility of oil in different solvents varies with temperature. This technique has been used for fractionation.
Data source location	Dehradun, India
Data accessibility	The data has been submitted to Data in Brief.
Related research article	NA

## Value of the data

• The article describes a novel, simple and cost-effective method for isolating lipid fractions from neem oil.
• The data provides an insight about effect of reduced temperature during lipid isolation, using hydrophobic and hydrophilic solvents. Hence, lipids from neem oil are separated based on their affinity towards solvent.
• The isolated fractions are different in composition and characteristics. This property can be utilized by several researchers in therapeutics.
• The oily lipids (NOC; NOM) can also be used as an excipient for encapsulating and targeting several drugs to desired site of action. Hence, different types of formulation approaches can be developed using isolated fractions [1], [2].

## 1 Data

**Fig. 1:**
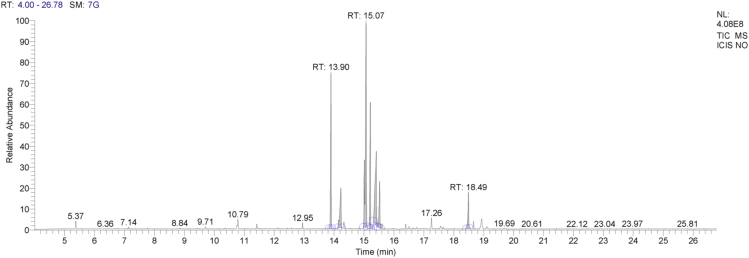
GC spectra of Neem oil (NO).

**Fig. 2:**
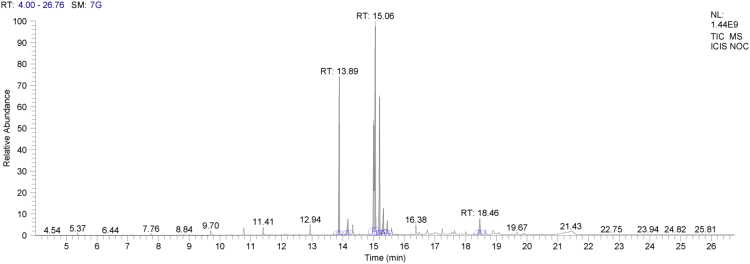
GC spectra of Neem oil chloroform (NOC) fraction.

**Fig. 3:**
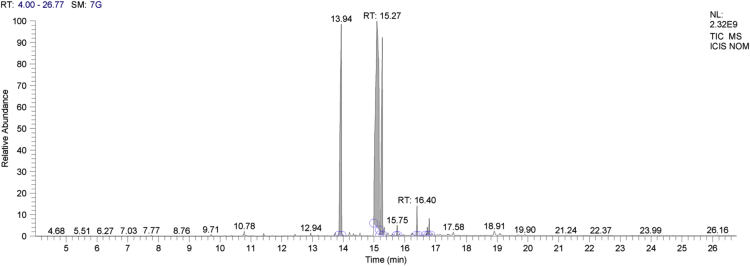
GC spectra of Neem oil methanol (NOM) fraction.

**Fig. 4:**
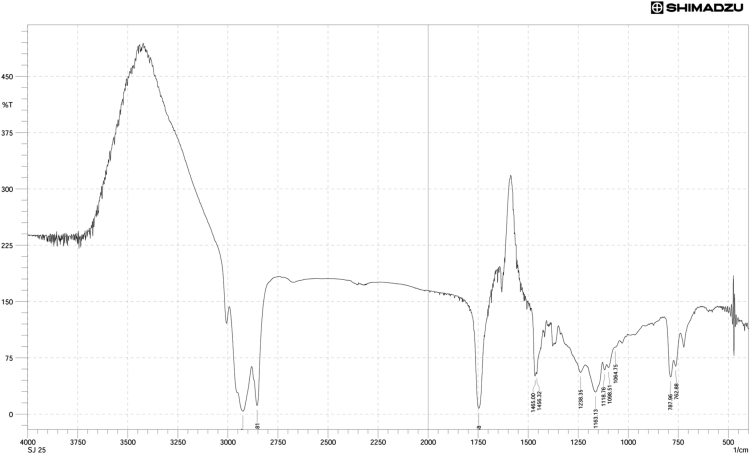
IR spectra of Neem oil (NO).

**Fig. 5:**
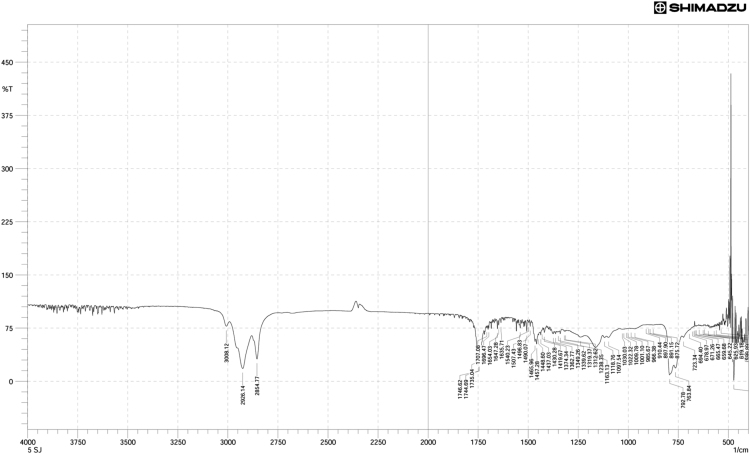
IR spectra of Neem oil chloroform (NOC) fraction.

**Fig. 6:**
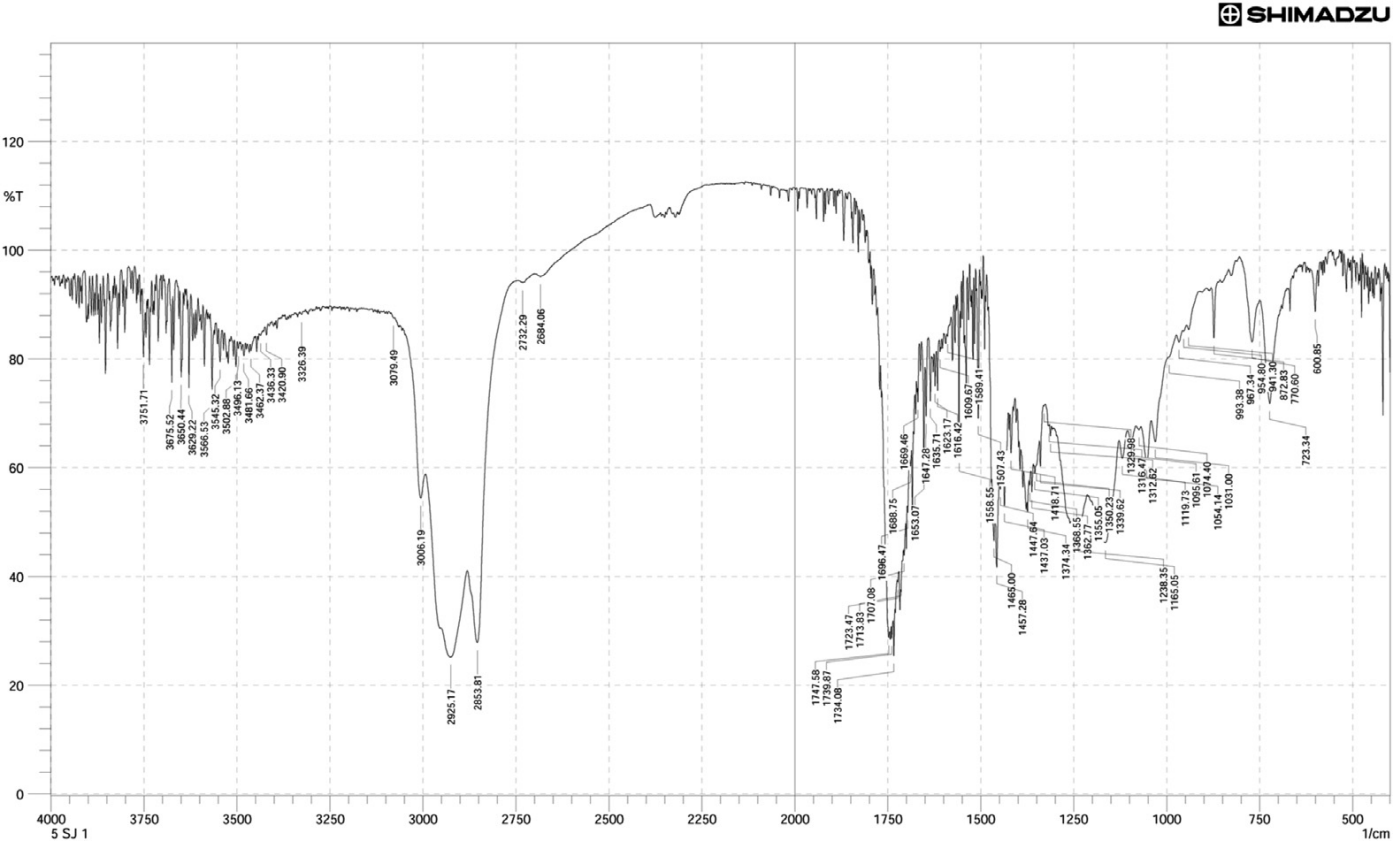
IR spectra of Neem oil methanol (NOM) fraction.

**Fig. 7:**
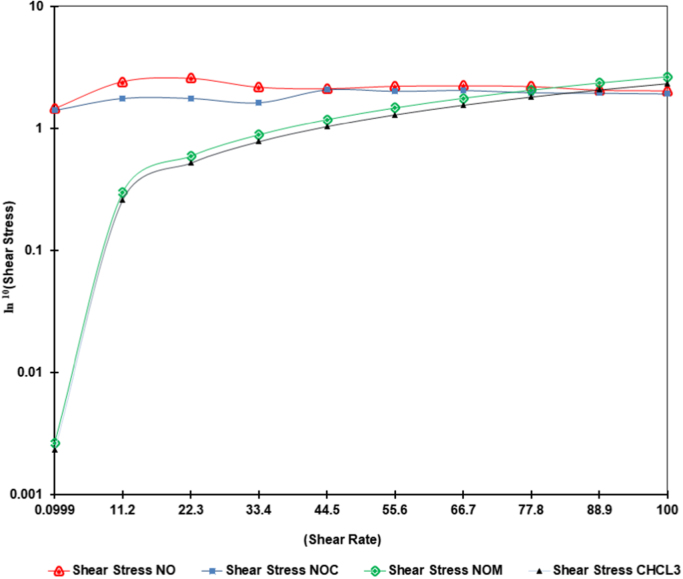
Graph showing shear rate comparison of Neem oil with its fractions and chloroform.

**Fig. 8:**
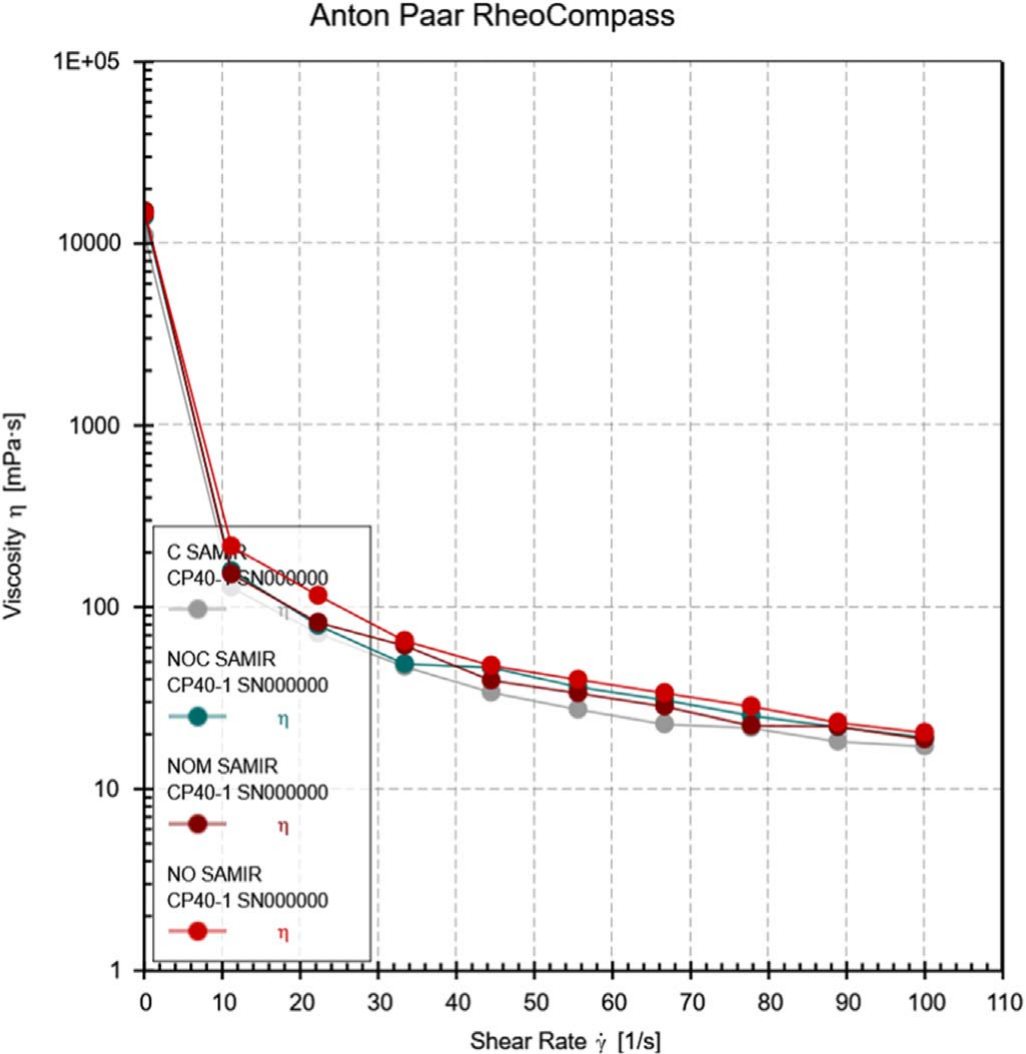
Viscosity comparison between Neem oil, its fractions and chloroform.

Neem oil, a medicinally and chemically complex lipid has a wide range of pharmacological activities [3], [4], [5], [6], [7]. The data obtained can be used for differentiating isolated fractions from neem oil. The Gas chromatograph and IR spectra assists in differentiating between chemical components of oil with its fractions. The data from GC chromatogram (Fig. 1, Fig. 2, Fig. 3) of neem oil and its fractions displays similar Retention times at 13.9 and 15.2, representing Hexadecanoic acid methyl ester and Methyl stearate. However, the compounds represented at these retention times of 13.9 and 15.2 shows difference in peak area (Table 1). The data from IR spectra also differentiates the Neem oil with its fractions (Fig. 4, Fig. 5, Fig. 6). The rheological differentiation of neem oil with its fractions (NOC; NOM) are clearly represented through Table 2. The data shows the difference in viscosity and shear stress of oils, diluting solvent with increasing shear rate (Fig. 7, Fig. 8). Hence the spectroscopic and rheological data of Neem oil and its fractions assists in their characterization and differentiation.

**Table 1:** RT (Retention times) of different compounds identified using GC spectra.

**Name of compound**	**Neem oil (NO)**		**Neem oil (Chloroform fraction – NOC)**		**Neem oil (Methanol fraction – NOM)**	
	**RT**	**Peak area**	**RT**	**Peak area**	**RT**	**Peak area**
**•** **Hexadecanoic acid, methyl ester**	13.9	16.43	13.89	20.22	13.94	23.87
**•** **1-(+)-Ascorbic acid 2,6-dihexadecanoate**	14.23	8.08				
**•** **9-Octadecenoic acid (Z)-, methyl ester**	15.07	33.88	15.06	49.21		
**•** **Methyl stearate**	15.21	13.42	15.2	18.26	15.27	17.78
**•** **6-Octadecenoic acid**	15.41	17.14				
**•** **Octadecanoic acid**	15.53	6.04	15.45	1.83		
**•** **Oleic acid, (2,2-dimethyl-1,3-dioxolan-4-yl) methyl ester**	18.49	5	18.49	2.93		
**•** **n-Hexadecanoic acid**			14.17	2.91		
**•** **cis-Vaccenic acid**			15.32	4.65		
**•** **cis-13-Octadecenoic acid, methyl ester**					15.09	55.54
**•** **Methyl 16-hydroxy-hexadecanoate**					15.75	0.5
**•** **Eicosanoic acid, methyl ester**					16.4	1.36
**•** **Methyl 15-hydroxy-9,12-octadecadienoate**					16.73	0.51
**•** **E,E,Z-1,3,12-Nonadecatriene-5,14-diol**					16.79	0.95

**Table 2:** Shear rate and viscosity of oils with diluent as estimated by Anton Par Rheometer.

**Time in [s]**	**Shear rate [1/s]**	**Shear stress in [Pa]**				**Viscosity in [mPa-s]**			
**Constant terms**		**(NOC)**	**(NOM)**	**(NO)**	**CHCL**_**3**_	**(NOC)**	**(NOM)**	**(NO)**	**CHCL**_**3**_
**60**	0.0999	1.41	1.5	1.4631	1.10	14,109.7	15,087.1	14,639.7	11,008
**120**	11.2	1.77	1.7	2.4258	1.44	158	151.8	216.6	128.3
**180**	22.3	1.77	1.84	2.5823	1.60	79.4	82.3	115.8	71.8
**240**	33.4	1.63	2.05	2.1826	1.58	48.8	61.5	65.3	47.2
**300**	44.5	2.08	1.76	2.1268	1.51	46.7	39.6	47.8	33.9
**360**	55.6	2.02	1.87	2.2256	1.52	36.4	33.6	40	27.3
**420**	66.7	2.06	1.9	2.2468	1.52	30.8	28.4	33.7	22.8
**480**	77.8	1.97	1.74	2.2137	1.69	25.3	22.3	28.5	21.7
**540**	88.9	1.95	1.96	2.0657	1.63	22	22.1	23.2	18.3
**600**	100	1.93	1.89	2.0438	1.72	19.3	18.9	20.4	17.2

## 2 Experimental design, materials, and methods

Neem oil (NO) has important role in drug delivery as a vehicle and in cosmetics. In our study the oil is fractionated to 2 fractions (NOC; NOM) using a simple cold-temperature based liquid–liquid extraction method. The selection and role of solvent plays an important role for separating the fractions from oil. Separation is potentiated at low temperatures as the inter-molecular interactions decreases within the solvents which converted a uniphase system to bi-phasic system.

The solvents used during experiment were chloroform, hexane and methanol (purchased from Merck India Ltd.). Chemicals used were NaCl (CDH India) and concentrated H_2_SO_4_ (Merck India Ltd.). Neem oil for fractionation was procured from local market. All other reagents were of analytical grade and were used as received.

### 2.1 Fractionation of Neem oil

Neem oil was dissolved in a solvent mixture of chloroform and methanol (Ratio – 2:1). The solvent ratio was optimized to ensure maximum extraction. The mixture was kept overnight at 4 °C for 12 h. The layers were separated carefully in cold conditions. All the fractions were heated over a water bath at controlled temperature of 20–30 °C to remove the solvents. The Chloroform fraction (NOC – 120 mL); Methanol fractions (NOM – 18 mL) were stored in dark.

### 2.2 Characterization of isolated oil fractions

#### 2.2.1 Preparation of fatty acid methyl esters for GC analysis

An acid catalyzed method was used to transform fatty acids to their methyl esters. 50 mg of oil was mixed with Methanol with 2% H_2_SO_4_, followed by addition of n-hexane to flask. The sample was refluxed for 45 min, followed by addition of saturated NaCl for separating the phases. The upper phase was collected and stored for GC analysis [8]. The investigation of isolated saponified extracts was performed on a GC–MS equipment (Thermo Trace 1300GC coupled with Thermo TSQ 800 Triple Quadrupole MS). **The Investigational conditions for GC–MS system were as follows**: Column used – TG 5MS (30 m × 0.25 mm, 0.25 µm; Thermo Fisher Scientific, Waltham, MA USA). Flow rate of carrier gas – 1 mL/min. In gas chromatography initial oven temperature − 60 °C (holding time kept at 2 min) was increased to 280 °C (holding time kept at 10 min) at 10 °C/min, with injection volume – 1 µL and temperature at 250 °C. The ion source was set at 230 °C. Samples were run at a mass range (m/z) between 50 and 600 *m*/*z*. The results were compared by using NIST/EPA/NIH Mass Spectral Library (NIST 08) and NIST Mass Spectral Search Program (Version 2.0f). The software used during our analysis was XCalibur 2.2SP1 with Foundation 2.0 SP1. However, the differences in composition of Neem oil and the fractions (NOC, NOM) separated using hydrophobic and hydrophilic solvents are shown through Table 1.

#### 2.2.2 FT IR of lipid extracts

FTIR spectrometer (Shimadzu FT IR Infinity-1, Japan) equipped with a DLATGS (deuterated L-alanine triglycene sulfate) pyroelectric detector and a KBr/germanium as beam splitter. It was connected to computer having IR-Solution software, which was used for obtaining FT IR data of isolated oil fractions. These spectra were recorded as % Transmittance (%*T*) values at each data point. The difference in location of band intensities (wavelength at *x*-axis) reveals presence/absence of functional groups in neem oil and its fractions.

#### 2.2.3 Rheology measurement and effect of viscosity with change in shear rates

Rheology measurements of the isolated oil extracts were performed using a stress-controlled Anton Paar MCR 72 rheometer (Anton Paar, Austria) in cone and plate geometry. The temperature of Rheometer was kept constant at 10 °C, while all adjustments were done before the measurements. Neem oil and its fractions were diluted with Chloroform (1:9) as the geometry required 1 mL of sample. The rheology of chloroform used, was also estimated in same geometry and under similar conditions. After achieving stability, the sample was sheared at varying shear rate from 0 s^−1^ to 100 s^−1^ for 10 min and then the shear stress vs. shear rate curve was obtained with 10 measuring points of 60 s duration. Similarly the effect of viscosity has been studied with changing shear rate from 0 s^−1^ to 100 s^−1^ for 10 min. Viscosity was measured on a logarithmically increasing scale. The reduction in temperature was required to avoid loss of chloroform during experiment at room temperature. Chloroform being a less viscous fluid has reduced effect on different fractions during viscosity measurements.
